# The effect of conventional versus electronic cigarette use on treatment outcomes of peri-implant disease

**DOI:** 10.1186/s12903-021-01784-w

**Published:** 2021-09-27

**Authors:** Reham AlJasser, Mohammed Zahid, Mohammed AlSarhan, Dalal AlOtaibi, Saleh AlOraini

**Affiliations:** 1 Present Address: Department of Periodontics and Community Dentistry, Dental College, King Saud University, PO Box 60169, Riyadh, 11545 Saudi Arabia; 2 Department of Preventive Dental Sciences, College of Dentistry, Prince Sattam Bin Abdulaziz University, Al Kharj, Kingdom of Saudi Arabia

**Keywords:** Smoking, Peri-implant disease, Risk factor, Peri-implantitis therapy

## Abstract

**Aim:**

To compare changes in clinical periodontal parameters (gingival consistency, colour, BOP, PI, PD) and changes of salivary inflammatory biomarkers (IL-1 β, IL-6, MMP-8, TNF- α and TIMP-1 between conventional, electronic cigarette smokers and non-smokers after peri-implant treatment.

**Methods:**

Study participants were grouped into three groups (i) Conventional cigarette smokers (ii) Electronic cigarette smokers and (iii) non-smokers respectively. A total of 60 adult patients aged (40–56 years) with 60 implants with active per-impantitis was included.Clinical and Biological parameters were evaluated before surgical treatment at baseline, one, six and twelve month post treatment. Pearson’s chi-square test was used to compare the distribution of the categorical while Two-way repeated analysis of variance was used to compare the mean values of quantitative outcome variables among all study groups across the 4 time points.

**Results:**

A total of 60 subjects (60 implants) were selected and classified into three groups as per their smoking method 20 participants in each group with one single targeted implant diagnosis with active peri-implantitis. The gingival colour, the change was statistically significant at one year of post treatment.The gingival consistency distribution across the three groups is not statistically significant at baseline, but it is statistically significant at one-month (*p* = 0.001), six months (*p* = 0.029) and at the completion of one-year (*p* = 0.018) post treatment. The plaque index of 100% of non-smokers had changed to ‘0’ and 35% change in cigarettes and 30% change in electronic smokers which is statistically significant (*p* = 0.016).The prevalence of BOP was observed in the three groups as 72%, 76.5% and 88.9% at baseline. The mean values of PD have shown statistically significant change across the three groups over the four-time intervals of observation (*p* = 0.024). The comparison of mean values of IL-1 β, IL-6 and TIMP-1 has shown statistically significant change across the three groups over the four intervals of observation (*p* < 0.0001).

**Conclusions:**

Electronic cigarette smoking was found to be most prevalent risk indicator for peri-implantitis. Compromised response of peri-implantitis treatment both clinically and biologically was found more among electronic cigarette smokers when compared to conventional cigarette smokers and non-smokers.

*Trial registration*: This case-control study was conducted at King Saud University’s Dental College, Riyadh, Saudi Arabia, in accordance with “Helsinki Declaration of Human Studies” and approved by the Institutional Review Board (Reference no: 87563).

## Background

Oral rehabilitation with dental implants confers functionally stable and successful outcomes in completely or partially edentulous patients. In contemporary dentistry, implant failure often results from loss of osseointegration between the implant and the peri-implant tissues. Major contributing factor in implant failure is the inflammatory change seen in response to the microbial plaque formation that can negatively affect the peri-implant tissues [1, 2]. According to a new scheme of periodontal and implant disease classification, peri-implant mucositis is defined as the reversible inflammation of soft tissue surrounding dental implants. Some common signs of peri-implant mucositis include redness, swelling, and bleeding on gentle probing. Whereas peri-implantitis is described as the irreversible inflammatory process due to degeneration of connective tissue between the bone and osseo-integrated oral implants that usually cause bone resorption and implant loss in advanced cases [3, 4]. The imbalance between the bacterial challenge and host response at the soft tissue–implant interface triggers this inflammatory process, predicted to be different from those observed around natural teeth in periodontal disease [5–8].

A recent published systematic review highlighted the strong negative impact of smoking and its direct association with severity of peri-implantitis [9]. Conventional tobacco cigarette use is one of the most common causative factors of peri-implantitis and periodontitis. The overall frequency of participants presenting with periodontitis has been significantly higher among smokers [10]. Besides, a robust overall correlation has been reported between smoking, tooth loss, and implant failure [11]. Furthermore, a prospective study showed a high degree of implant failure in patients who are heavy smokers [12].

In 2004, Electronic cigarette (E-cigarette) was introduced as a new version of nicotine delivery system [13]. A study suggested that E-cigarette is less cytotoxic than the tobacco cigarette [14], while another study indicated that menthol in E-cigarettes causes similar harm to periodontal ligament fibroblast as compared with conventional cigarettes [15]. All chemicals in tobacco products despite the form or usage include carbon monoxide, hydrogen cyanide, and other compounds with high carcinogenicity. Since the concentration of nicotine in both conventional and E-cigarettes is similar it causes comparable vasoconstrictive activity on gingival blood vessels concomitant with resultant damage on gingival fibroblasts [16].

Since peri-implantitis is an inflammatory process, the detection of salivary biomarker levels in peri-implant soft tissue, peri-implant sulcular fluid (PISF) or saliva were found to be valid additive diagnostic tools to detect the peri-implant disease activity [17, 18]. Certain biomarkers when isolated from active sites of peri-implantitis showed statistical differences when compared to healthy stable peri-implant sites [19]. These include matrix metalloproteinase-8 (MMP-8) (Collagenase-2) which is the main enzyme responsible for extracellular collagen matrix degradation and tissue inhibitor metalloproteinase-1 (TIMP-1) which is induced by pro-inflammatory cytokines (IL-1, IL-6, OSM, LIF, and TNF-α) [20].

An imbalance of MMP and TIMP production correlates with pathological conditions including peri-implant diseases. Several investigations reported that MMP-8/TIMP-1 ratio in both saliva and PISF can be a predictable sign of active hard tissue destruction around implant that can be faster than in the area around a periodontitis lesion [21, 22]. Other important inflammatory markers of an active process includes Interleukin-1β (IL-1β). A strong correlation was found between high levels of IL-1β and the progression of peri-implantitis when measured at different sites [23]. Similarly, high concentration of interleukin-6 (IL-6) appears in peri-implantitis cases which was correlated with the active phase of bone resorption [24]. On the other hand, the inhibitor of matrix metalloproteinase-1 (TIMP-1) can be used as a detection tool for disease regression since it was proven to be in higher concentrations in sites exhibiting signs of periodontal and peri-implant tissue health [25]. Therefore, successful dental implant treatment depends on considering all the above stated factors which, if managed, can contribute towards the wellbeing of patient’s oral and peri-implant health.

E-cigarettes are becoming a popular smoking method globally. The prevalence of E-cigarette use among young adults (age range: 16–30 years) has been studied in several international regions, including the USA, Canada, Europe, and New Zealand. Data from these regions showed that E-cigarette use increased rapidly over the past few years, reaching as high as 14.7–50% of overall smokers [16–19]. Thus, the effects of smoking newly developed e-cigarettes on various medical and dental conditions including peri implant diseases and their treatment outcomes need to be understood more closely.

The rationale of this prospective study was to evaluate smoking as a high-risk factor for peri implant diseases. This study aims to compare conventional cigarette smokers, electronic smokers and non-smokers treatment outcomes and risk indicators of peri-implantitis diseases as there is a lack of such studies in the existing literature.

The objectives of this study were: 1-To compare changes in clinical periodontal parameters between C-smokers and E-smokers with non-smokers after peri-implant treatment. 2-To evaluate changes in salivary biomarkers after treating peri-implantitis and compare responses among C-smokers and E-smokers with non-smokers.

## Methods

### Study design and sample collection

This case–control study was conducted at King Saud University’s Dental College, Riyadh, Saudi Arabia, in accordance with “Helsinki Declaration of Human Studies” and approved by the Institutional Review Board (Reference No: 87563).

The study protocol followed “STROBE – Strengthening the Reporting of Observational studies in Epidemiology” guidelines for preparing the manuscript. The sample size was calculated based on G-Power software based on a previous pilot study. Altogether 60 adult patients aged 18–70 years with 60 implants were examined both clinically and radiographically at the Department of Prosthodontics and data were collected from June 2018 to September 2019. Study participants were grouped into three groups (i) Exclusive C-smokers (ii) Exclusive E-smokers and (iii) Never smokers (non-smokers).To avoid bias each group was assigned 20 participants, the study sample was calculated using “Raosoft” sample size calculator (Raosoft inc. USA) and the data from preliminary studies. Patients who met the inclusion criteria were informed about study objectives. After signing informed consent patients were enrolled in the study. It was ensured that the enrolled subjects in the conventional smokers and electronic smokers’ group had a record of daily smoking to be categorized into respective groups. During study, all patients with implants underwent surgical treatment to control active peri-implantitis.

### Inclusion criteria

The patients whose implant surgery and prosthetic restorations were performed in the same institute were included in this study. The study inclusion criteria were based on three categories: (i) patients with history of conventional cigarette smoking for past 1 year (C-smokers), (ii) E-smokers/vapers with history of E-cigarette use over past 1 year, (iii) non-smokers with no tobacco consumption history but with peri-implantitis. It was mandatory in all groups that patients underwent at least one implant surgery during the past 2 years.

Patients who received conventional-length (> 6 mm), non-turned, 2- and 3-piece titanium implant* (Straumann® BLX Implant System, Institute Straumann AG, Peter Merian-Weg 12CH-4002 Basel, Switzerland) and those suffering from peri-implantitis of one or more implants replaced by single crown implant-supported restoration with a minimum period of six months of functional occlusal loading observed during the appointment for dental examination were included in the study.

## Exclusion criteria

All other conditions including (I) water pipe smokers, (II) smokeless tobacco users, (III) patients with medical history of systemic, blood, renal, cardiovascular, diabetes, immunodeficiency, all levels of osteoporosis, and metabolic disorders (IV) antibiotic use for a medical or dental reason within the last 12 months (1 year) before the examination (V) intake of medicaments that could have an effect on turnover of the bone and mucosal healing (i.e., anti-inflammatory medications, steroids, antiresorptive therapy) (VI) pregnant and lactating mothers (VII) absence of baseline radiographs taken at the time of placement of the implant or final crown (VIII) active periodontal disease on natural teeth (IX) lack of ability or refusal to sign the informed consent.

### Peri-implant disease diagnosis

All enrolled patients were screened according to “Classification of periodontal and peri-implant diseases and conditions—2017, work group IV consensus report” [26] including conditions as follows: (i) with ≥ 6 mm probing depth (ii) in tissue level implant bone level ≥ 3 mm apical either at smooth-rough interface or implant coronal portion (iii) on gentle probing suppuration or bleeding initiates. The clinical examination also included demographic details, smoking habits to determine their classification into either of the three test groups i.e., C-smokers, E-smokers, and non- smokers. Final peri-implant disease status was determined based on the degree of peri-implantitis severity. This severity grading is based on patient’s degree of bone loss ratio and depth of defect measured at implant neck. (i) Grade i: less than 25% of implant length (3–4 mm) (ii) Grade ii: in-between 25 to 50% of implant length (4–5 mm) (iii) Grade iii: more than 50% of implant length (> 6 mm). Bone morphology and severity in all peri-implantitis cases were classified according to Monje et al. [27] as Cl II, which is defined as mostly being supracrestal/horizontal defects.

All assessments were made at quarterly intervals in all the three groups. Peri-implant bone loss was assessed through radiographic evaluation for implants with PD > 6 mm. Intra-oral radiographs were taken through dental X-ray machine with digital radiographic analysis through imaging software “Sidexis XG, Sirona, Germany” program.

### Treatment protocol

All the included cases were re-evaluated for non-surgical treatment protocol to control inflammation before surgical therapy was decided. Afterwards they were scheduled for surgical open flap debridement of implant site that consisted of submarginal incision, full thickness flap reflection, granulation tissue removal, and implant surface modification along with supporting bone recontouring without the need of grafting.

### Primary outcomes

The primary outcomes in the present study were assessed by two types of parameters that are presented as follows:

The clinical parameters included periodontal probing depth (PD), bleeding on probing (BOP), Plaque index (PI), gingival color (GC), gingival consistency (GCC), and keratinized tissue width (KTW). For biological parameters, MMP-8 (Neutrophil collagenase), Interleukin-6 (IL-6), Interleukin-1β (IL-1β), and TIMP-1 were taken into consideration.

### Clinical parameters

The following clinical parameters were assessed using a plastic probe (11 Colorvue Probe, Hu-Friedy) at each implant site: PD was measured by inserting the probe within the sulcus area with gentle pressure (less than 0.25 Ncm) exerted at three points of each buccal and lingual surfaces around the neck of an implant along with a probe placed parallel to the implant crown titled 10 degrees inward at the proximal points [16]. BOP was assessed either by the presence (+) or absence (−) of bleeding at the site of probing immediately after periodontal pocket depth measurement [12]. PI was assessed either by the presence (+) or absence (−) of plaque on four surfaces (mesial, distal, palatal and buccal) of a crown after placing disclosing agent. The measurements of PD and BOP were performed at six surfaces per implant: mesio-buccal, mid-buccal, disto-buccal, mesio-lingual/palatal, mid-lingual/palatal, and disto-lingual/palatal. GC and GCC were evaluated via direct visual assessment, i.e., visibility of the periodontal probe. The GC for subjects was estimated through use of “spectroradiometer PR-670 with MS-75 accessory lens (Photo Research, Chatsworth, CA)”. The color identification of the gingiva as either pink or red was done through “CIE ∆L*∆a*∆b*” color parameters representing ∆L = Lightness, ∆a and ∆b (Chroma). These tooth layer parameters are adjusted to induce dark to light color scheme. On average three measurements were used to define color difference using ∆Eab* and ∆E00. The minimum and maximum color difference for ∆E00 was 0.25 & 30.38 units and for ∆Eab were 0.41 and 33.34 units respectively. The threshold of GC change was in accordance with the acceptability threshold of 50:50 “color differences that were accepted by 50% of the observers” [13]. KTW was determined by the presence of or absence of ≥ 2 mm of keratizined tissue. It was measured from the peri-implant marginal mucosa to the muco-gingival junction (MGJ) at the buccal and lingual marginal portion of the peri-implant’s mucosa. Finally, standardized periapical radiographs were taken at the time of clinical examination with the long cone paralleling method and film holders (Rinn XCP, Dentsply Corporate, York, PA, USA) and for bone level confirmation compared with a baseline radiograph taken at the time of prosthesis placement [28]. Remarkably, the radiographs were scanned to obtain standardized digital images with a resolution of 1200 dpi. These digital images were imported and analyzed using a specialized computer software (ImageJ v 1.49, Research Services Branch, National Institute of Health, Bethesda, MD, USA). The pixel/mm ratio was calibrated using the implant length as a fixed reference to compensate for potential radiographic distortion. For the bone loss evaluation, the radiographic distance between the implant shoulder level and the most coronal bone-to-implant contact level was calculated mesially and distally, parallel with the long axis of the implant.

### Saliva sample collection

Patients were instructed not to eat or drink for at least three hours prior to saliva collection. To minimize fluctuations in salivary secretion related to circadian rhythm, all collections were performed at a fixed time of the day. They were instructed to relax and swallow all saliva present in their mouths 5 min before starting the saliva collection. While seated and leaning forward, they were asked to spit all the saliva they produced into a graduated test tube over a period of five minutes. Furthermore, all surgical procedures and clinical measurements as well as saliva sample collections were taken by a single investigator (R.A.) to exclude possible operator-dependent bias at baseline, and the time intervals of 1 month, 6 months and 1 year of post peri-implantitis treatment.

### Biological parameters and enzyme-linked immuno-sorbent assays

Saliva samples were collected to evaluate the relative amount of MMP-8 (Neutrophil collagenase), IL-6, IL-1β, and TIMP-1. Enzyme-linked immunosorbent assay (ELISA) was used to study the levels of different biomarkers (Elabsciences ®, Houston, Texas, USA). ELISA results were read at 450 nm by using a micro-plate reader (Bio-Rad Laboratories Inc., Hercules, CA). ELISA was performed in accordance with the manufacturer's instructions [30, 31]. ELISA kit uses Sandwich-ELISA as the method. The micro-ELISA plate provided in the used kit (Elabscience) has been pre-coated with an antibody specific to Human MMP-8, IL-6, IL-1 β, and TIMP-1. Standards samples were added to appropriate micro-ELISA plate wells and combined with the specific antibody. Then biotinylated detection antibodies specific for Human testes proteases and Avidin-Horseradish Peroxidase (HRP) conjugate was added to each micro-plate successively and incubated. After incubation, free components were washed away.

The substrate reagent was added to each well, only those wells that contained MMP-8, IL-6, IL-1 β, TIMP-1, biotinylated detection antibody, and Human Avidin-HRP conjugate appeared blue in color. The enzyme–substrate reaction was terminated by adding stop solution and a change in colour to yellow was shown for the final solution. The optical density (OD) was measured by using Biotek Synergy HT the micro-plate reader (Synergy HT, Biotek, Vermont, USA) at a wavelength of 450 nm ± 2 nm. The OD value is proportional to the concentration of biomarkers. The concentrations in samples were calculated by comparing the OD of the samples with the standard curve [29, 30].

### Maintenance visits

The primary objective of maintenance visits was to improve reduction rate of peri‐implantitis and attain peri‐implant marginal bone stability through proper follow-up visits. Patients were contacted and asked to undergo clinical and radiographic examination at the time intervals of 1, 6, and 12 months (1 year) post treatment as a part of maintenance care plan. As infection control has significant impact on peri-implant diseases, detailed oral hygiene instructions and professional oral hygiene treatment was provided to all patients by clinician. The trans-mucosal implant portion already surrounded by the keratinized immovable and attached tissue during placement of implant (one stage implant placement) or during abutment connection (two stage implant placement) was taken into consideration [30]. Maintenance plan indicated that if peri-implant PD was ≤ 3 mm with no BOP and visible plaque, no treatment was needed for patient on follow-up visit. However, if peri-implant PD was ≤ 3 mm but bleeding on probing or plaque was present then the patient was counselled for maintaining oral hygiene through proper brushing, use of interdental brushes, dental floss, and plaque control measures were introduced. Instruments softer than titanium i.e., plastic based scaling instruments, dental flossing, polish with paste and rubber cup were used for implant cleaning.

If peri-implant PD was ≥ 3 mm, patients were evaluated for bone loss determination, if there was no bone loss with absence of plaque formation and BOP, no treatment was recommended to the patient. However, if an observation of no bone loss with presence of plaque and BOP was made, then the patients were advised to follow oral hygiene guidelines and local debridement and redo of surgical resection procedure if required. Under this condition, if PD was between 4 and 5 mm resulting from tissue swelling, it was corrected by peri-implant plaque control measurements. In case, PD was ≥ 5 mm due to presence of pus, it required topical antiseptic preparation (combination of 3% hydrogen peroxide W/V or 2% Chlorhexidine) application.

Finally, the Maintenance care plan included oral hygiene instructions, implant cleaning, local and open debridement, and use of systemic antibiotics for patients.

### Data analysis

Descriptive statistics (mean, standard deviation, frequencies, and percentages) were used to describe the categorical and quantitative outcome variables. Pearson’s chi-square test was used to compare the distribution of the categorical variables among the three study groups at each point of follow-up for each group comparison. Two-way repeated analysis of variance was used to compare the mean values of quantitative outcome variables among the three study groups across the four time points (baseline, 1 month, 6 months and 1 year) of follow-up. A *p*-value of ≤ 0.05 was used to report the statistical significance of the results. Statistical analysis was carried out using SPSS 24.0 version (IBM Inc., Chicago USA) statistical software.

## Results

### Demographic parameters

A total of 60 patients with a total 60 bone level implants were selected as per their smoking method, each of the 20 participants with one affected implant in each test group was distributed by same evaluator to avoid biasness. The sex distribution was approximately even across the three groups. The mean age was higher in C-smokers 54.1 years (11.5) in comparison to E-smokers with mean age 46.8 years (11.4) and non-smokers 46.9 years (12.8). The implant site was evenly distributed across the groups, with most common implant site was that of tooth number 26 in all the three groups; 4 (20%) in C-smokers, 5 (25%) in E-smokers, and 4 (20%) in non-smokers. All implants included were bone-level implant type. The implant size of 4.1 × 10 mm was frequently used across the three groups; 13 (65%) in E-smokers, 12 (60%) in C-smokers and non-smokers. The number of cigarettes smoking per day was found as 9.2 (0.6) for conventional smokers and 6.5 (0.9) for electronic smokers respectively (Table 1). No significant difference was found between groups among previously mentioned demographic and clinical variables. The mean use of cigarette packs per year in C-smoker group was 30 packs per year. (Table 1).

**Table 1:** Characteristics of study subjects with their method of smoking

Characteristics	Cigarettes smoker (n = 20)	Electronic smoker (n = 20)	Non-smokers (n = 20)
	Mean (SD)	Mean (SD)	Mean (SD)
Age (years)	54.1	46.8	46.9
**Sex**			
Male	12 (60)	9 (45)	10 (50)
Female	8 (40)	11 (55)	10 (50)
No. of Cigarette smoked daily	9.2 (0.6)	6.5 (0.9)	**–**

Implant site	n (%)	n (%)	n (%)
14	2 (10)	2 (10)	2 (10)
15	2 (10)	3 (15)	2 (10)
16	2 (10)	2(10)	3(15)
25	1(5)	0	0
26	4 (20)	5 (25)	4 (20)
34	2 (10)	1 (5)	1 (5)
35	2 (10)	2 (10)	2 (10)
36	2 (10)	2 (10)	2 (10)
45	1 (5)	1 (5)	2 (10)
46	2 (10)	2 (10)	2 (10)
Total	20	20	20

Implant size	n (%)	n (%)	n (%)
4.1 × 10 mm	12 (60)	13 (65)	12 (60)
4.1 × 12 mm	2 (10)	3 (15)	2 (10)
4.8 × 10 mm	4 (20)	0	3 (15)
3.3 × 8 mm	0	2 (10)	2 (10)
3.3 × 8 mm	2 (10)	2 (10)	1 (5)
Total	20	20	20

### Clinical parameters

**Fig. 1:**
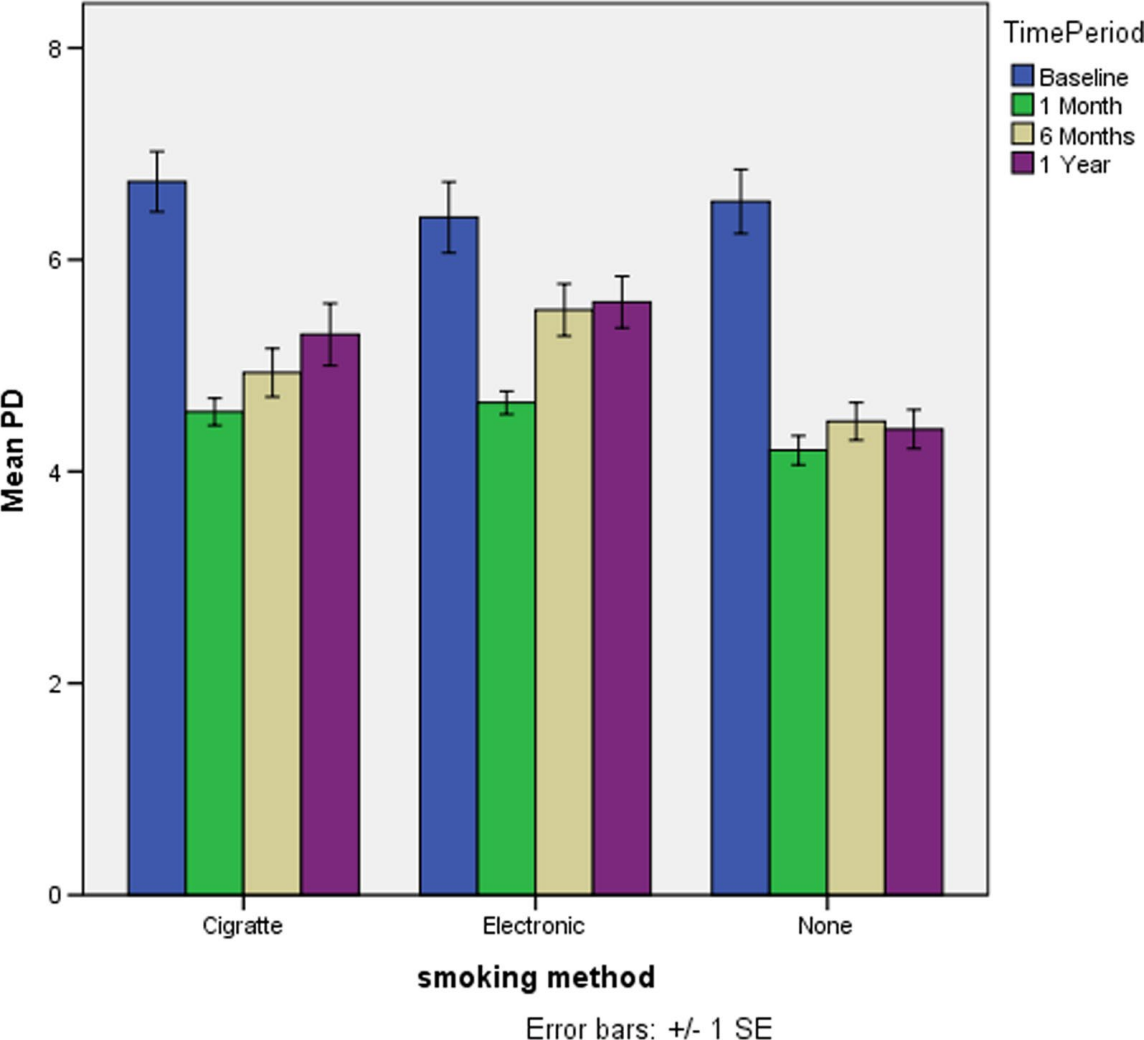
Mean change in Pocket Depth (PD) among 3 study groups in four time periods or evaluation

The comparison of outcome variables among the three groups at each of the four observations conducted after specific time intervals are presented in Table 2. The GC was red in all the 20 subjects of three groups at baseline, whereas it had changed to pink in 90% of C- smokers, 55% of E- smokers and 100% in non-smokers at one month follow-up. Similar pattern was observed at six months and at one year of post treatment. In regards to GC, the change was statistically significant at one year of post treatment, where the change from red to pink is significantly higher in C-smokers and non-smokers in comparison with E-smokers (*p* = 0.043) (Fig. 1).

**Table 2:** Comparison of categorical outcome variables in relation to the smoking type over 4 time points of study follow-up period

Outcome variables	Time period and smoking type											
	Baseline			1 month			6 months			1 Year		
Gingival color	Cig. F (%)	E F (%)	None F (%)	Cig. F (%)	E F (%)	None F (%)	Cig. F (%)	E F (%)	None F (%)	Cig. F (%)	E F (%)	None F (%)
Red	20 (100)	20 (100)	20 (100)	2 (10)	9 (45)	0	4 (21)	7 (37)	2 (10)	5 (25)	9 (45)	2 (10)
Pink				18 (90)	11 (55)	20 (100)	15 (79)	12 (63)	18 (90)	15 (75)	11 (55)	18 (90)
χ^2^-value; *p*-value	–			–			–			6.307; 0.043*		
Gingival consistency	14 (70)	10 (50)	17 (85)	2 (10)	9 (45)	0	4 (21.1)	9 (47.4)	2 (10)	5 (25)	10 (50)	2 (10)
Edematous firm	6 (30)	10 (50)	3 (15)	18 (90)	11 (55)	20 (100)	15 (78.9)	10 (52.6)	18 (90)	15 (75)	10 (50)	18 (90)
χ^2^-value; *p*-value	5.70; 0.058			14.92; 0.001			7.06; 0.029			8.04; 0.018*		
**Plaque index**												
0	9 (45)	7 (35)	0	16 (80)	13 (65)	20 (100)	15 (79)	13 (68.4)	18 (95)	16 (80)	13 (65)	19 (95)
1	11 (55)	13 (65)	20 (100)	4(20)	7 (35)	0	4 (21)	6 (31.6)	1 (5)	4 (20)	7 (35)	1 (5)
χ^2^-value; *p*-value	11.42; 0.003				8.24; 0.016			–			–	
**BOP**												
Yes	13 (72)	13 (76.5)	16 (88.9)	3 (15)	2 (10)	0	5 (26.3)	9 (47.4)	2 (10.5)	6 (30)	10 (50)	2 (10)
No	5 (28)	4 (23.5)	2 (11.1)	17 (85)	18 (90)	20 (100)	14 (73.7)	10 (52.6)	17 (89.5)	14 (70)	10 (50)	18 (90)
χ^2^-value; *p*-value		1.64; 0.441			–			–		7.62; 0.022*		

*Statistical significant *p* < 0.05

A statistically significant change was observed in C-smokers and non-smokers, where 70% of edematous gingiva at baseline in C-smokers had changed to 'firm' in 90% at one-month duration, 78.9% after six months and 75% at the completion of one year. Whereas 85% of edematous at baseline in non-smokers had changed to 'firm' in 100% at one month, 90% after six months and 90% at the completion of one-year. One of the determining factors for this change from edematous to firm gingiva was the decrease in pro-inflammatory cytokines levels among non-smokers and a decreased number of peri-implantitis associated micro-biota due to pathogens presence in implants that may colonize in periodontal pockets of teeth over the period of year was observed in non-smokers than in C-smokers and E-smokers. The difference was statistically significant at one month, (*p* = 0.001), after six months (*p* = 0.029), and at the completion of one year (*p* = 0.018). The PI distribution (0 vs 1) across the three groups had shown statistically significant difference at baseline, where 100% of non-smokers had PI 1, when compared with other two groups (55% & 65%) who had PI of 1 (*p* = 0.003). But significant change was observed at one month, when the PI of 100% of non-smokers had changed to ‘0’ and 35% change in cigarettes and 30% change in E-smokers which is statistically significant (*p* = 0.016). Similar pattern of change was observed after six months and one year of post treatment. The prevalence of BOP was observed in the three groups as 72% among C-smokers vs 76.5% among E-smokers and 88.9% in non-smokers at baseline; however, at one month it had reduced to 15%, 10%, and 0% (respectively). A significant increase was observed in all three groups after one year of the treatment (*p* = 0.022) (Table 2).

The comparison of clinical parameters among the three groups across four-time intervals is shown in Table 3. However, no statistically significant difference in relation to the mean values of KTW was observed. The mean values of PD have shown statistically significant change across the three groups over the four-time intervals of observation. The mean values of PD had significantly reduced from baseline to one month and this reduction maintained the same pattern at six months and one-year post treatment, except for E-smokers group where the mean PD values increased after six months and one year. Both the time period and its interaction with smoking type are statistically significant. (*p* < 0.0001; *p* = 0.024) (Fig. 1).

**Table 3:** Comparison of quantitative outcome variables in relation to the smoking type over 4 time points of study follow-up period

Outcome Variables	Time period and smoking type^#^—Mean (Sd.,)											
	Baseline			1 month			6 months			1 Year		
	Cig	E	None	Cig	E	None	Cig	E	None	Cig	E	None
KTW (mm)	1.7 (0.9)	1.9 (1.4)	1.9 (1.1)	1.7 (.9)	1.9 (1.4)	1.9 (1.1**)**	1.8 (1.1)	1.7 (1.3)	1.9 (1.1)	1.7 (0.9)	1.9 (1.4)	1.9 (1.1)
F-value; *p *value	Time period: 0.006; 0.941 smoking type: 0.207; 0.814											
PD (mm)	6.9 (0.9)	6.3 (1.4)	6.5 (1.3)	4.4 (.5)	4.7 (.5)	4.2 (0.6)	4.7 (0.6)	5.5 (1.1)	4.5 (0.8)	4.8 (0.9)	5.6 (1.1)	4.4 (0.8)
F-value; *p *value	Time period: 39.98; *p* < 0.0001 smoking type: 2.53; *p* = 0.024*											
MMP8 (pg/ng)	20.1 (6.3)	27.1 (5.3)	9.4 (4.3)	10.1 (1.6)	11.4 (1.8)	3.3 (0.8)	14.9 (16.3)	22.9 (35.2)	5.2 (4.6)	12.2 (5.6)	16.9 (7.9)	4.1 (2.2)
F-value; *p *value	Time period: 8.52;* p* < 0.0001 smoking type: 0.78; p = 0.583											
IL6 (pg/ng)	153.7 (20.6)	183 (8.4)	47.2 (3.7)	63.2 (11.3)	85.6 (6.4)	15.6 (2.3)	65.4 (15.6)	96.3 (34.5)	18.6 (10.6)	80.6 (43.3)	120.6 (50)	19.4 (11.1)
F-value; *p* value	Time period: 127.82; *p* = 0.0001 smoking type: 10.86; *p* = 0.0001*											
IL1 β (pg/ng)	3.55 (0.06)	3.62 (0.08)	2.84 (0.04)	1.96 (0.10)	2.14 (0.11)	1.28 (0.17)	2.01 (0.09)	2.44 (0.44)	1.32 (0.19)	2.30 (0.62)	2.84 (0.75)	1.47 (0.49)
F-value; *p*-value	Time period: 168.95; *p* < 0.0001 Time period smoking type: 26.38; *p* < 0.0001*											
TIMP-1	4.61 (3.4)	1.8 (2.4)	6.9 (2.0)	25.6 (16.8)	14.2 (4.8)	49.8 (19.6)	10.6 (5.4)	5.9 (4.1)	42.3 (25.6)	10.1 (5.8)	5.7 (4.1)	43.6 (24.4)
(pg/ng)												
F-value; *p *value	Time period: 42.44; *p* < 0.0001 smoking type: 10.12, *p* < 0.0001*											

Test:Two-way repeated analysis of variance

^#^Cig. = Cigarettes; E = Electronic; None = No smoking

^##^Log_10_ values due skewness

*Statistical significance: *p* < 0.05

### Biochemical parameters

The mean values of MMP-8 at baseline for the three groups had significantly reduced when compared with those at 1 month in all the three groups with a marginal increase at 6 months and 1 year. However, the change was not statistically significant in the mean values of MMP-8 for all the three groups when its baseline levels were compared to the levels at other three time points. Also, for E-smokers, significant difference in the mean values of MMP-8 at the three different time intervals (1 month, 6 months and at the completion of 1 year) was observed in comparison with the C-smokers and non-smokers but with no significant difference.

The mean values of IL-6 have shown statistically significant change across the three groups when observed at the given time intervals. The mean values of IL-6 had significantly reduced from baseline to one month, following a similar pattern after 6 months and 1 year. Findings indicated statistically significant association between the time period and its interaction with smoking type (*p* < 0.0001; *p* < 0.0001).

The comparison of mean values of IL-1β has shown statistically significant change across the three groups over the four intervals of observation. The mean values of IL-1β had significantly reduced from baseline to one month and the reduction of values was consistent after six months. However, the mean values of IL-1β increased at the completion of one year but was still significantly lower than the baseline values. Both the time period and its interaction with smoking type are statistically significant (*p* < 0.0001; *p* < 0.0001).

**Fig. 2:**
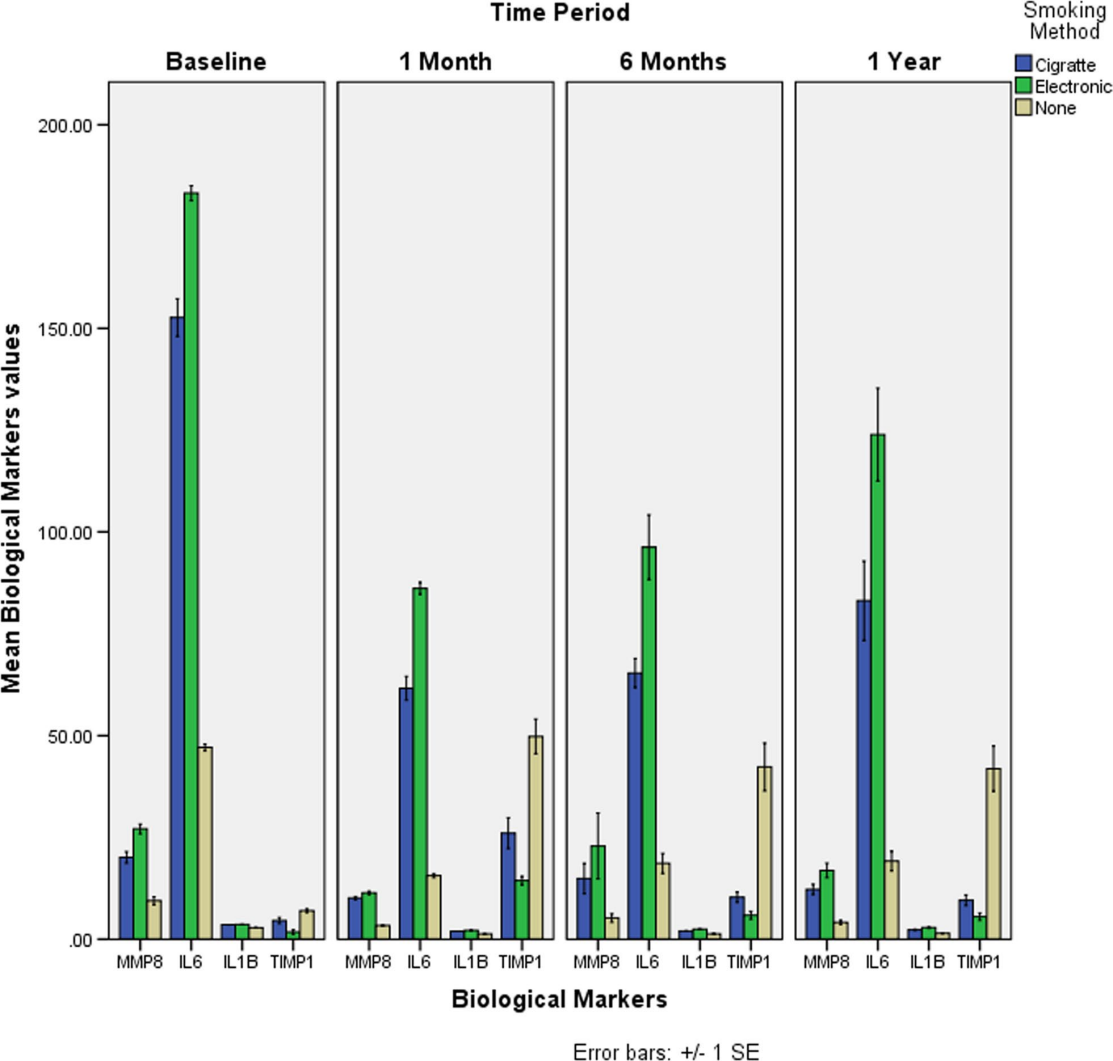
Mean changes of salivary inflammatory biomarkers among 3 study groups in four time periods or evaluation

The comparison of mean values of TIMP-1 has shown statistically significant change across the three groups during the four-time intervals. Across all the three groups, the increase in the mean values TIMP-1 from baseline was substantial but the increase of TIMP-1 values in non-smokers was significantly higher at all the three intervals when compared to its base line values. A statistically significant association was found between the time period and smoking type (*p* < 0.0001; *p* < 0.0001) (Table 3) (Fig. 2).

## Discussion

The present study compared clinical changes between C-smokers and E-smokers with non-smokers, along with the evaluation of salivary inflammatory biomarker changes among patients treated for peri-implantitis. Prior studies outlined the role of cigarette consumption as a risk factor for peri-implantitis and reported conflicting results [31–38]. Recent evidence found that smoking, among various other cofactors, has a negative impact on clinical outcomes and peri-implant lesion reduction [32, 33]. It further stated smoking as an essential risk indicator for surgical peri-implantitis treatment success [39, 40].

However, findings in this study showed that short-term outcomes were comparable at one-month post-therapy. However, at one-year follow-up, non-smokers had favorable results when compared to both groups of smokers. Interestingly, E-smokers showed persistent inflammatory outcomes compared with C-smokers in GC and GCC. This unexpected finding may suggest that specific components in these new innovative types of smoking methods may be responsible for reduced blood flow and inflammatory responses in comparison with conventional cigarettes as supported by previous observations provided by de Waal et al. [41].

As for plaque accumulation, the PI was higher in the group of non-smokers in comparison to the other two groups at baseline. Nevertheless, the significant clinical improvement observed in the three groups noted through 1 month, decreased slightly after 6 months and 1 year follow-up but was maintained lower than the baseline status. The prevalence of BOP showed improvement as well after the treatment. However, it was noticed that these changes were more among non-smokers compared to other smoking groups due to the vasoconstrictive effect of nicotine and other smoke by-products as well as heat on blood vessels and capillaries [38]. Finally, mean PD reduced among non-smokers and C-smokers in all the time points, while in E-smokers, this reduction had relapsed after 6 months.

In regards to salivary biomarkers which can give an assessment of the systemic biologic response to different levels and fluctuations of inflammation, MMP-8, IL-1β and IL-6 have shown statistically significant decrease after one month of treatment from the baseline. However, their values raised marginally at 6 month and 1 year after the treatment with further increase among E-smokers followed by C-smokers and non-smokers. This observation is in line with several previous studies, as for instance, Al-Sowygh et al. [42] in a case–control study reported higher levels of MMP-8 in smokers when compared to non-smokers. Furthermore, in another case–control study by Abduljabbar et al. [43] a significantly increased level of IL-1β and IL-6 in the peri-implant sulcus fluid was observed among smokers in comparison to the non-smokers [43]. Whereas, all study groups revealed increased values of TIMP-1 throughout the treatment period and demonstrated the highest values among non-smokers and least among E-smokers. Further, these values again showed some relapse through long term follow up, especially in the E-smokers.

These changes in biological markers confirm the clinical presentations among the three groups along the period of the study, proving that non-smokers group has the best response to peri-implantitis treatment compared to other groups. Results evident in this study confirm that the major source of inflammation and infection is controlled, and preferable biological and clinical response is usually predicted [40]. Moreover, when major risk factors are present such as smoking, the expected treatment outcomes can be more challenging to attain optimal success. Several previous studies had elaborated that even among smokers, successful immediate results can be shown post-treatment of peri-implantitis [38].

Upon authors knowledge, this study is the first study to compare peri-implantitis surgical treatment outcomes among C-smokers and E-smokers. Since E-smoking is a new and widely spread method among people, the investigations related to the effect of this type of smoking on the periodontium and implant supporting structures are important. Therefore, it is worthwhile to provide investigational efforts to understand how this new type of smoking can act among inflammatory cascades. Moreover, the response in regards to peri-implant and periodontal tissue is to achieve proper treatment protocol and prevention for E-smokers receiving dental implants.

Limitations of the present study include relatively small sample size in each group as well as a lack of information from certain participants regarding the frequency of smoking, as well as types of E-cigarettes. In addition, Not using peri-implant sulcular fluid (PISF) and relying only on saliva to evaluate changes in biological markers. Therefore, future recommendation include the conduction of trials over a large sample size, along with the inclusion of diverse demographic and racial groups to understand different responses and to compare different types of electronic smoking methods and the use of PISF or peri-implant tissue samples to evaluate changes of biological markers among participants more accurately. The above recommendations are further important to understand the variations among different products and to have more understanding of their by-products. Finally, it is suggested to compare different approaches to peri-implant treatment based on different peri-implantitis stages and related types of defects to achieve a cornerstone of favorable treatment approach that can achieve long term success.

## Conclusion

Among patients suffering from peri-implantitis, E-smokers have shown least favorable long term clinical and biological outcomes related to treatment in comparison to cigarette smokers and non-smokers, the later showed the best long-term outcomes after 1 year follow-up. All clinical parameters showed improvement after initiating the therapy in comparison to baseline. Salivary biomarkers (MMP-8, IL-1β and IL-6) have shown a decline in all groups treated. However, their values increased marginally at six months and one year with further increase among E-smokers followed by cigarette smokers followed by non-smokers. On the other hand, the TIMP-1 levels showed consistent increase in all groups throughout the treatment.

## Data Availability

The datasets used and analyzed during the current study are available from the corresponding author on reasonable request.

## Abbreviations

E-Cigarette: Electronic cigarette
BOP: Bleeding on probing
PD: Probing depth
KTW: Keratinized tissue width
IL-1 β: Interleukin 1 beta
IL-6: Interleukin 6
MMP-8: Matrix metalloproteinase
TIMP-1: Tissue inhibitor of metalloproteinase
