# Correction to: The effect of conventional versus electronic cigarette use on treatment outcomes of peri-implant disease

**DOI:** 10.1186/s12903-022-02141-1

**Published:** 2022-04-01

**Authors:** Reham AlJasser, Mohammed Zahid, Mohammed AlSarhan, Dalal AlOtaibi, Saleh AlOraini

**Affiliations:** 1 Department of Periodontics and Community Dentistry, Dental College, King Saud University, PO Box 60169, Riyadh, 11545 Saudi Arabia; 2 Department of Preventive Dental Sciences, College of Dentistry, Prince Sattam Bin Abdulaziz University, Al Kharj, Kingdom of Saudi Arabia

## Correction to: BMC Oral Health (2021) 21:480 https://doi.org/10.1186/s12903-021-01784-w

Following publication of the original article [1], the affiliation of the author “Mohammed Zahid” was incorrectly published as “Saudi Board of Periodontics Resident, Ministry of Health, Riyadh, Saudi Arabia.” The correct Affiliation is “Department of Preventive Dental Sciences, College of Dentistry, Prince Sattam Bin Abdulaziz University, Al Kharj, Kingdom of Saudi Arabia.”

The original article has been corrected.
